# Editorial

**Published:** 1913-08

**Authors:** 


### EDITORIAL

The Business Office of the *Journal-Record* is 23 West Alabama Street.

The Editorial Office is 1014-15 Atlanta National Bank Building.

Address all Business Communications to *Journal-Record of Medicine,* 23 West Alabama Street.

Make remittances payable to *The Journal-Record of Medicine.*

On matters pertaining to the Editorial and Original Communications, address Edgar G. Ballenger, M. D., Atlanta, Ga.

Reprints of Original Articles will be furnished at cost price. Requests for the same should always be made in THE MANUSCRIPT.

We will present, postpaid, on request to each contributor of an original article, twenty (20) marked copies of *The Journal-Record of Medicine* containing such article.

### ENFORCEMENT OF OUR NEW MEDICAL PRACTICE LAW.

At last, after much work and worry, we have secured the passage of a most valuable "Medical Practice Bill," which is soon to become a law. This bill at first had much opposition, but in spite of it there were only four votes against it in the Senate and three in the House, which shows the sentiment, in the main, was and is in favor of such a bill. All thoughtful physicians and laymen realized full well the need of such a law. It provides a composite board of medical examiners, with five regulars, three eclectics and two homeopaths. The board has the right to decline to examine applicants who show diplomas from colleges which do not come up to the required standards and which are not sufficiently equipped to provide adequate laboratory courses in chemistry, bacteriology, etc. The minimun entrance requirements to medical colleges are fixed at 14 Carnegie Units. Thus must the standard of medical education and training be raised, and medical colleges must either give sufficient training in the study and treatment of disease or close their doors and cease their imposition both upon the students and the public.

The law clearly defines the practice of medicine, but does not include osteopaths, and Christian Scientists; the latter though are not allowed to charge for their alleged services. Avertising fakirs and quacks will leave because their advertising must discontinue and their practice cannot stand alone. It means better days in Georgia both for the patients and physicians.

In order that this bill may be productive of the desired and possible good, its rigid enforcement should be secured from the start so that it may command the wholesome respect of those who may be inclined to perpetrate infringements. The local and state medical associations should lend their influence and finances toward this purpose: if further funds are necessary to proceed with rigorous enforcement of this law, we should raise subscriptions from the doctors and force every trial in a business-like manner to show the public that the physicians of Georgia are determined to raise their standard and protect the public.

---

### THIRTEENTH ANNUAL SESSION OF THE CHATTAHOOCHEE VALLEY MEDICAL AND SURGICAL ASSOCIATION. HELD IN MONTGOMERY, ALABAMA, TUESDAY AND WEDNESDAY, JULY 15-16, 1913.

Meeting called to order by Dr. Geo. M. Niles, Atlanta, Ga. President..

Invocation by Rev. F. D. Devall, Montgomery, Ala.

Address of welcome by Hon. W. A. Gunter, Jr., President City Commissioners of Montgomery, who spoke in part, as follows:

"A minister is ever welcome to our homes and we turn to him in joy and in trouble for consolation, but it is perhaps to the physician and surgeon we turn most often; when taken suddently sick, or when accidents occur, it is then that we quickly turn to our good friends the doctors Montgomery is glad to welcome the Chattahoochee Valley Medical and Surgical Association; we are always glad to have with us such men as you are, not only because her people are so deeply inbued with the spirit of respect for men of your calling, but because we are proud of our town and want to show it off; we have the greatest town in the country, and we think we have the greatest town in the world. We know that we have one of the cleanest towns in the world, and almost daily we are receiving communications asking us for copies of our sanitary ordinances and laws, some of which have originated in Montgomery and all of which have been gone over very carefully and studied systematically to glean from them the best that can be gotten from them. All over this city we have tremendous mains that lead throughout every section of the city, and we believe that in six or eight months from now we will have one of the most perfect sewerage systems to be found anywhere. We know we have a beautiful city and we want you gentlemen to look around while you are here and go back home and talk about our city and its many advantages.

Gentlemen, again I want to welcome you. Anything we have is yours while you are here; we turn the city over to you and all we ask is for you to go in and have a good time. Gentlemen, I thank you.

(Applause)

Dr. Niles said, "We greatly appreciate Mr. Gunter's kind words, and I want to say so far as the repute of Montgomery is concerned, in regard to beauty, hospitality, etc., I assure you that you are entirely true as to her repute.

The medical profession is nothing if not progressive, and the time has passed when the man who was known as a moss-back had any place in this profession; we have a man in this Association who is an expert and therefore cannot be classed as a moss-back; therefore, I am glad to call on this gentleman to make a little welcoming speech for the Montgomery County Medical and Surgical Association; I scarcely feel that he needs any introduction, but, gentlemen, I am glad to introduce to you the man that took the Moss out of moss-back. (Laughter and Applause)

Dr. Phillip B. Moss of Montgomery, responds:

"In behalf of the Montgomery County Medical and Surgical Association, it is my duty and great pleasure to welcome the members of the Chattanoochee Valley Medical and Surgical Association to our City. As the guests here of our 60 odd members, I wish to say that we are more than glad to have with us the followers of a profession reaching into the very life of the nation itself and whose sole purpose in the organization of its members, is for the study and research for the benefit and healing of our people. Gentlemen, we welcome you, and hope your stay in our city will be both beneficial and enjoyable. (Applause).

In the absence of Dr. J. M. Poer, West Point Ga., the President calls upon Dr. E. C. Thrash, Atlanta, Ga., who makes the followingresponse.

"Gentlemen, I am sorry that I did not know that Dr. Poer was going to be kept away at the very last minute or I would have gotten a little speech from him, but as I did not, I feel just a little bit embarrassed; I have made very many speeches and I believe that this is the first time I have been called upon to make an address of welcome or respond to one; but I can say this much; we are ready to receive what you have to give us. (Laughter) Our physiogomies will indicate to you that we are in a receptive mood; in fact, we have already received a sample of your hospitality; I received some of it this morning with my face buried in a sea of mist; I enjoyed it very much indeed because we have nothing like it now in Atlanta—I have been looking forward to it for a week or ten days. I started away from home with some apparatus I intended demonstrating here, but broke some of the bottles and wired for others; I was talking to a Montgomery man about it and showed him the telegram I received in response to my wire, and the telegram read: "Your bottles put in the express office at 7:25." He says: "You are all off; you don't need any bottles shipped here." I told him that Atlanta being always dry, eternally dry, that we got in the habit of getting our bottles ready in advance and carrying them with us. I want to tell you a little story that happened just before prohibition went into effect in Georgia. The Medical Association convention was held that time at Macon; that was about three years ago, and the members of the Atlanta Association had sent down quite a number of cases of beer, and as usual, our president was on the job; for better distribution and convenience they had the beer in a room at the hotel and we had to take turns at being tapsters; there was one old fellow who had been in there several times, and he came back again; I happened to be in there and this old fellow came in with his eyes all bleared and his moustache wet—in fact, he did not give it time to get dry between drinks, and he asked for another drink. Dr. Niles gave it to him, and handed him a card with words something like this: "I am Dr. Niles of Atlanta; I want to give you one of my cards." The old fellow kind of fished down in his pocket and said, "We, I already got two or three of them things now." "That's all right," said Dr. Niles, "I will just take those up and give you a fresh one." He is always ready; you can't lose him. Now, I am going to tell one on myself; some years ago when I had just commenced practicing, I was located in a little remote town; one of my neighbors used to drive to town with a two-horse wagon, and before he drove back he usually filled himself and his wagon up; well, one day coming home he drove his wagon into a ditch, the wagon body turned over and caught him by the neck under the wagon; somebody came for me and I went on down there, felt his pulse, looked wise, and prescribed a dose of calomel; I did not know any more about a broken neck—in fact, there were a lot of things I did not know anything about and did not find out until some years later. I left some instructions about what to do; the neighbors were all standing around talking about Mr. Treadwell's condition, and he had an Irishman who was superintendent on his farm. This Irishman had his share of Irish in him, and the people were all standing there talking about Mr. Treadwell, and George said; "Well, you can all stand here and talk if you want to about what to do for Mr. Treadwell, but I am going to get Dr. Brantley; I aint going to fool on that dam-long-legged Dr. Thach; he don't know a gut from a vein." (Laughter.)

I want to tell you another little story on Dr. Terrell; he was in Greenville and I was practicing in a little adjoining town; I had extracted three teeth from an old negro woman and charged her $1.50 for it and was trying to get her to pay me the $1.50. She said: "My gracious! Does you charge $1.50 for taking out them three teeth?" I told her yes and that I could not do it for any less. She said "Well I wisht I had er knowed dat; I kin go up to Greenville and get Dr. Terrell to take out them teeth and he would not charged me but 50 cents and he is a plum good doctor, too." (Laughter)

Now, gentlemen, I want to say to you that we are glad to be with you, and if you will just give us the chance, we are going to show you just what a fine time we can have. You will not see scientific knowledge scintillating from us, nor will you see sparks of brilliancy flying, but if you will just give us a chance we will show you how good a time we can have in this beautiful town of yours; our body-cells are receptors strong. I saw a little drawing before I came here which showed the receptors radiating in all directions and this made me think of how we were receptors of your hospitality; it applied to us as we are ready to receive all of the hospitality that you are willing to give. This is the first time I have ever been in Montgomery and I want to say that I am delighted with your city; it is clean; its streets are broad; it is perfectly beautiful; it seems that the city was designed before it was built, not like Atlanta, having the appearance of being built around cow trails. We thank you much for your hearty welcome. (Applause)

Dr. Baker: I would like to say just a few words before they leave; the Entertainment Committee has arranged that the visiting members are to be given tickets to the vaudeville performance which commences at 9 o'clock; the Reception Committee wil see that every member has a ticket. The entertainment is to be at the Empire Theatre on Commrce St., just below the Exchange Hotel. I also wish to say for Mr. Gunter and the physicians of Montgomery that the Beauvoir Club is open to them; by the way, after the vaudeville performance,e ther is to be a Dutch lunch served on the roof garden of the Beauvoir Club; we hope that every one of you will be there; I also want to say that the Country Club, Beauvoir, or any other of our clubs are open to you; all you have to do is to go and call for what you want and you will get it; they are wide open; the latch string is on the outside to you while you are in Montgomery. We hope you will enjoy yourselves.

The next place of meeting was appointed as West Point, Georgia.

The following officers were elected for the ensuing year:

President, Dr. J. M. Poer, West Point, Ga.

1st Vice-President, Dr. H. S. Monroe, Columbus, Ga.

2nd Vice-President, Dr. W. W. Stevenson, Roanoke, Ala.

Secretary-Treasurer, Dr. W. J. Love, Opelika, Ala.

Board of Council

President, Dr. J. H. McDuffie, Columbus, Ga.

Dr. A. L. Harlan, Alexander City, Ala.

Dr. H. B. Disharoon, Roanoke, Ala.

Dr. Geo. H. Cooper, Opelika, Ala.

Dr. Hugh McCullogh, West Point, Ga.

Program Committee

Chairman, Dr. Hansell Crenshaw, Atlanta, Ga.

Dr. H. S. Monroe, Columbus, Ga.

Dr. H. W. Terrell, LaGrange, Ga.

Dr. L. W. Johnson, Tuskegee, Ala.

Dr. W. J. Love, Opelika, Ala.

After a vote of thanks had been passed to the Montgomery County Medical Association for the use of the Association rooms during the Convention, the Association adjourned sine die.

